# ﻿The seventh species of the newt genus *Tylototriton* in Thailand: a new species (Urodela, Salamandridae) from Tak Province, northwestern Thailand

**DOI:** 10.3897/zookeys.1215.116624

**Published:** 2024-10-15

**Authors:** Porrawee Pomchote, Parada Peerachidacho, Wichase Khonsue, Pitak Sapewisut, Axel Hernandez, Chitchol Phalaraksh, Parunchai Siriput, Kanto Nishikawa

**Affiliations:** 1 Department of Biology, Faculty of Science, Chulalongkorn University, Bangkok 10330, Thailand; 2 Research Institute for Languages and Cultures of Asia, Mahidol University, Nakhon Pathom 73170, Thailand; 3 Department of Biology, Faculty of Science, Chiang Mai University, Chiang Mai 50200, Thailand; 4 LASER, College of Biology & the Environment, Nanjing Forestry University, Nanjing 210000, China; 5 Department of Environmental Sciences, Faculty of Sciences and Technics, University Pasquale Paoli of Corsica, Corte 20250, France; 6 Department of National Parks, Wildlife and Plant Conservation, Bangkok 10900, Thailand; 7 Graduate School of Global Environmental Studies, Kyoto University, Kyoto 606–8501, Japan; 8 Graduate School of Human and Environmental Studies, Kyoto University, Kyoto 606–8501, Japan

**Keywords:** Conservation, crocodile newt, morphology, phylogeny, *Tylototritonsoimalai* sp. nov.

## Abstract

A new species of the crocodile newt genus *Tylototriton* from Doi Soi Malai located at Mae Tuen Wildlife Sanctuary, Tak Province, northwestern Thailand is described based on molecular and morphological evidence, and named as *Tylototritonsoimalai***sp. nov.** The new species is the seventh recorded species of the genus *Tylototriton* reported in Thailand. It differs morphologically from its congeners by a combination of the following morphological characteristics: head longer than wide; snout blunt or truncate; sagittal ridge on head narrow, short and distinct; dorsolateral bony ridges on head pronounced and rough; parotoids distinct; vertebral ridge prominent, wide and not segmented; 14–16 distinct, rounded and isolated rib nodules but posterior nodules connected; tips of fore- and hind limbs overlapping when adpressed along the body. The body background color is black, while the color markings are orange. Molecular analysis indicated that *Tylototritonsoimalai***sp. nov.** is a distinct lineage and sister to *T.uyenoi* with a 4.1% genetic sequence divergence based on the mitochondrial NADH dehydrogenase subunit 2 gene. The new species is currently restricted to the hill evergreen forests of Doi Soi Malai. The implementation of a strategic plan is recommended to protect both the species and its habitat from anthropogenic activities.

## ﻿Introduction

The salamandrid genus *Tylototriton* Anderson, 1871, also known as crocodile newts, currently contains 40 nominal species with several unnamed taxa endemic to Southeast Asia and ranging across the eastern Himalayas, central to southern China (including Hainan island), and to the northern parts of Indochina (Wang et al. 2018; Pomchote et al. 2021b; Dufresnes and Hernandez 2022; Frost 2024). The genus has been divided into three subgenera: *Tylototriton*, *Yaotriton* Dubois & Raffaëlli, 2009, and *Liangshantriton* Fei, Ye & Jiang, 2012 (e.g., Nishikawa et al. 2013a; Pomchote et al. 2021b). This genus includes cryptic species that are difficult to distinguish based solely on external morphological characteristics (Pomchote et al. 2021b). Consequently, molecular phylogenetic methods, combined with extensive morphological investigations, are necessary to assess the taxonomic status of the genus *Tylototriton*. These approaches have led to the description of several new species of crocodile newts (Frost 2024).

**Figure 1.**
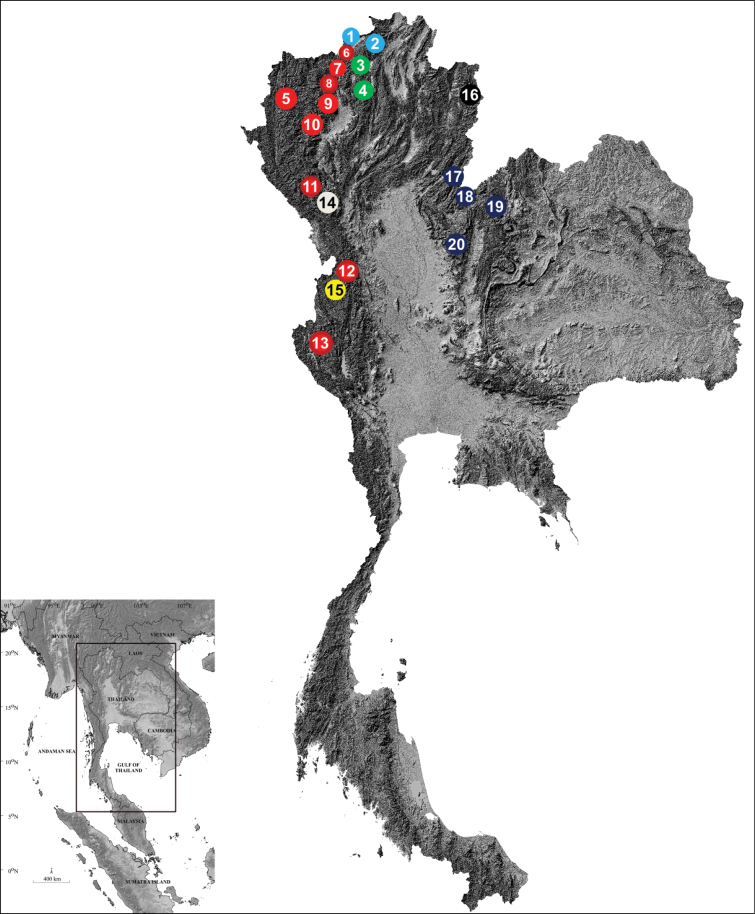
Current distribution of the genus *Tylototriton* in Thailand. *Tylototritonverrucosus* (pale blue): **1** Doi Pha Hom Pok NP, Chiang Mai Province **2** Doi Chang, Chiang Rai Province; *T.anguliceps* (green): **3** Si Dong Yen, Chiang Mai Province **4** Khun Chae NP, Chiang Rai Province; *T.uyenoi* (red): **5** Namtok Mae Surin NP, Mae Hong Son Province **7** Doi Ang Khang, Chiang Mai Province **9** Doi Suthep, Chiang Mai Province **10** Doi Inthanon, Chiang Mai Province; T.cf.uyenoi (red outlined with dark red): **6** Doi Mak Lang, Chiang Mai Province **8** Chiang Dao WS, Chiang Mai Province **11** Doi Mon Jong, Chiang Mai Province **12** Mae Wong NP, Kamphaeng Phet Province **13** Khao Laem NP, Kanchanaburi Province; *T.soimalai* sp. nov. (tan): **14** Doi Soi Malai, Mae Tuen WS, Tak Province; *T.umphangensis* (yellow): **15** Umphang WS, Tak Province; *T.phukhaensis* (black): **16** Doi Dong Ya Wai, Doi Phu Kha NP, Nan Province; and *T.panhai* (dark blue): **17** Phu Soi Dao NP, Uttaradit Province **18** Phu Suan Sai NP, Loei Province **19** Phu Luang WS, Loei Province **20** Phu Hin Rong Kla NP, Phitsanulok Province. NP = National Park and WS = Wildlife Sanctuary. The map is modified from https://www.mitrearth.org.

In Thailand, six species of *Tylototriton* from the subgenera *Tylototriton* and *Yaotriton* are currently known: *T.verrucosus* Anderson, 1871; *T.uyenoi* Nishikawa, Khonsue, Pomchote & Matsui, 2013; *T.anguliceps* Le, Nguyen, Nishikawa, Nguyen, Pham, Matsui, Bernardes & Nguyen, 2015; *T.phukhaensis* Pomchote, Khonsue, Thammachoti, Hernandez, Suwannapoom & Nishikawa, 2020; *T.umphangensis* Pomchote, Peerachidacho, Hernandez, Sapewisut, Khonsue, Thammachoti & Nishikawa, 2021 (all belonging to the subgenus Tylototriton); and *T.panhai* Nishikawa, Khonsue, Pomchote & Matsui, 2013 (subgenus Yaotriton). Among these six species, *T.uyenoi* displays various phenotypes with allopatric distribution in scattered and isolated mountainous areas (Nishikawa et al. 2013a; Hernandez 2016; Hernandez and Pomchote 2021). In the past, this species was identified as *T.verrucosus* Type I based on coloration and distribution range (Pomchote et al. 2008). Subsequently, morphological and molecular evidence showed that the Type I could be separated into two groups, *T.uyenoi* (Nishikawa et al. 2013a) and *T.anguliceps* (Le et al. 2015). According to Nishikawa et al. (2013a), *T.uyenoi* was originally distributed in three mountains, Doi (meaning “mountain” in Thai) Ang Khang, Doi Inthanon, and Doi Suthep, all in Chiang Mai Province, in northern Thailand. Subsequently, this species has been reported in new distribution locations across northern to western parts of Thailand: Namtok Mae Surin National Park (NP), Mae Hong Son Province, northern region (Pomchote et al. 2020a); Chiang Dao Wildlife Sanctuary (WS) (Michaels 2014), Doi Mon Jong, and Doi Mak Lang (Hernandez et al. 2019), Chiang Mai Province, northern region; Doi Soi Malai in Mae Tuen WS, Tak Province, northwestern region (Hernandez 2017); Umphang in Tak Province, western region (Hernandez et al. 2019); Mae Wong NP, Kamphaeng Phet Province, western region (Pomchote et al. 2021a); and Khao Laem NP, Kanchanaburi Province, western region (Hernandez and Pomchote 2020b) (Fig. 1). However, among the aforementioned reports, only the study by Pomchote et al. (2020a) included morphological variations and molecular analyses of *T.uyenoi* based on ten specimens from Mae Hong Son Province. Additionally, the study by Pomchote et al. (2021b) collected four crocodile newts from Umphang, Tak Province, and examined their morphology and genetics. The results indicated that the crocodile newts from Umphang belong to a lineage distinct from the known *Tylototriton* species, and they were described as a new species, *T.umphangensis*. In the other studies, newts were identified as *T.uyenoi* based on external morphology of one individual and its distribution, due to several reasons such as lack of permission to collect specimens or tissues, or the newts being found accidentally.

On 16 July 2014, Hernandez found an adult male crocodile newt in a muddy water pond located in a dipterocarp and mixed deciduous forest on the top of Doi Soi Malai, at an elevation of approximately 1,500 m a.s.l., in Mae Tuen WS, Tak Province, and tentatively assigned the specimen as *T.uyenoi* without conducting a detailed study (see Hernandez 2017). However, the male crocodile newt shown in figure 1 of this publication showed some morphological differences from the original description of *T.uyenoi* (see Nishikawa et al. 2013a). For example, the shape of the midsagittal ridge in the former is narrow, short, and distinct, while in the latter, it is indistinct. Additionally, the rib nodules in the former are connected posteriorly, whereas they are separated posteriorly in the latter. Furthermore, in 2015, the Tourism Authority of Thailand (TAT) confirmed the presence of *Tylototriton* in this locality by publishing pictures and a short VDO clip on MGR Online. The crocodile newt was identified using the Thai local name Ka-taang or Kra-taang (meaning lizard), or Jing-jok Nam (meaning house gecko and water, respectively) without providing its scientific name.

Thus, the aforementioned data lead to new field surveys being conducted at Doi Soi Malai, Mae Tuen WS where a newt population was discovered in a mud puddle on the road near the summit of the mountain. Detailed phylogenetic and morphological analyses of this population were performed to clarify its taxonomic status, and revealed that the specimens from Doi Soi Malai, Mae Tuen WS belong to a distinct lineage within the subgenus Tylototriton. Herein, we describe this population as a new species, *Tylototritonsoimalai* sp. nov.

## ﻿Materials and methods

### ﻿Sampling

**Figure 2.**
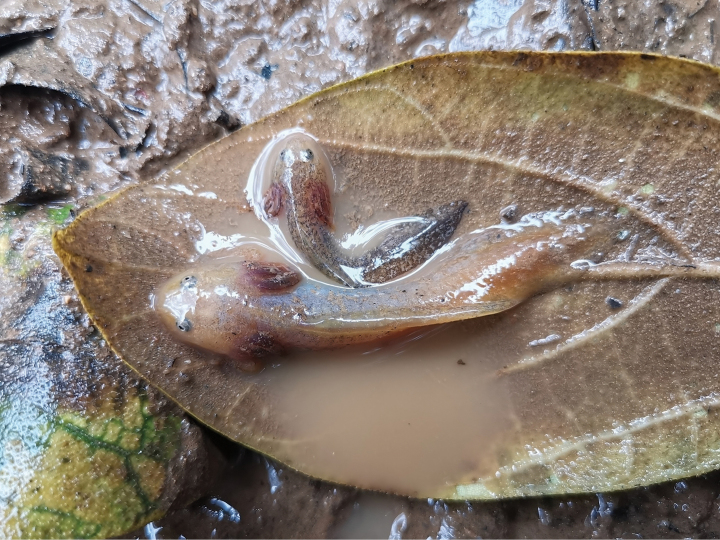
The two larvae of *Tylototritonsoimalai* sp. nov. in life.

The field survey was conducted on the 31 August 2022 at Mae Tuen WS, Tak Province, northwestern Thailand (Fig. 1, locality 14) using the visual encounter survey method (Heyer et al. 1994). Three newts were found during the daytime only in one mud puddle on the road through the highest peak of Doi Soi Malai, at ca 1,500 m a.s.l. Due to the turbid and muddy water, the newts were captured using an aquatic dip net and kept in plastic bags for examination. All newts were checked for sex and maturity, based on their cloacal characters (Pomchote et al. 2008). They were subsequently identified as breeding males. These male specimens were used for molecular and morphological analyses. Additionally, two larvae were discovered and photographed in this mud puddle (Fig. 2); however, while we were attempting to take morphological measurements, they managed to escape and hide in the puddle. Due to the discovery of the larvae, this mud paddle is considered a breeding site for the Mae Tuen WS newts.

Following previous studies (Pomchote et al. 2021b), live specimens were anesthetized by immersion in a solution of tricaine methanesulfonate (MS-222; 5 g/L) for ~ 5 min, then euthanized in a solution of chloretone (Heyer et al. 1994), and measured for morphometrics and body weight (BW) (see details below). The tissue samples (liver) of each specimen were taken, and subsequently stored in 95% (v/v) ethanol for molecular analysis prior to preservation. The voucher specimens were fixed in 10% buffered formalin, stored in 70% (v/v) ethanol, and then deposited in the Chulalongkorn University Museum of Natural History (**CUMZ**), Bangkok, Thailand.

### ﻿Molecular analyses

Total DNA was extracted from the liver using a PureDireX^TM^ genomic isolation kit (Bio-Helix, Taiwan). The mitochondrial NADH dehydrogenase 2 gene (ND2) was amplified using the polymerase chain reaction (PCR) with the SL-1 (5′–ATAGAGGTTCAAACCCTCTC–3′) and SL-2 (5′–TTAAAGTGTCTGGGTTGCATTCAG–3′) primers (Wang et al. 2018). Each PCR reaction consisted of 15 µL of OnePCR^TM^ Ultra (GeneDirex, Taiwan), which is a premixed solution, 1.5 µL of each primer (10 µM), 9 µL of UltraPure^TM^ DNase/RNase-Free distilled water (Invitrogen, USA), and 3 µL of DNA template. The thermal cycling was performed at 94 °C for 4 min, followed by 35 cycles of 94 °C for 30 s, 55 °C for 1 min, and 72 °C for 90 s (Wang et al. 2018). The PCR products were checked by agarose gel electrophoresis to confirm their size and estimate the concentration. The desired PCR products were purified and commercially sequenced by Bioneer Inc. in South Korea.

We combined the three new ND2 sequences of the Mae Tuen WS samples obtained in this study with those of the other related species available from GenBank (Table 1). We then constructed phylogenetic trees by Bayesian inference (BI) and maximum likelihood (ML) analyses using MrBayes v. 3.2.6 (Ronquist et al. 2012) and RAxML-NG v. 1.0.2 (Kozlov et al. 2019), respectively. The optimum substitution models were selected using Kakusan 4 (Tanabe 2011) for BI and ModelTest-NG v. 0.1.7 (Darriba et al. 2020) for ML. The codon-proportional model with the Hasegawa-Kishino-Yano-1985 (HKY85) model + Gamma (G) for each codon position for the BI and criterion used for model selection was AIC, with the codon-equal-rate model with the general time reversible model (GTR) + I + G being selected for ML. The BI analysis was performed as two independent runs of four Markov chains for 10 million generations, sampling one tree every 100 generations and calculating a consensus topology for 10,000 trees after discarding the first 25,001 trees (burn-in = 2,500,000). For the BI, we considered posterior probabilities (bpp) of 95% or greater as significant support (Leaché and Reeder 2002). The robustness of the ML tree was tested using bootstrap analysis (Felsenstein 1985) with 1,000 replicates, and we accepted tree topologies with bootstrap support (bs) values of ≥ 70% to be significantly supported (Huelsenbeck and Hillis 1993). Pairwise comparisons of uncorrected sequence divergences (*p*-distance by 2,842 base pairs; bps) were calculated using MEGA v. 7 (Kumar et al. 2016).

**Table 1.** Specimens of *Tylototriton* and other related species used for the molecular analyses in this study. CAS = California Academy of Sciences; CIB = Chengdu Institute of Biology; CUMZ (A) = Natural History Museum of Chulalongkorn University Section Amphibians; KIZ = Kunming Institute of Zoology; KUHE = Graduate School of Human and Environmental Studies, Kyoto University; MVZ = Museum of Vertebrate Zoology, University of California, Berkeley; NMNS = National Museum of Natural Science, Taiwan; VNMN = Vietnam National Museum of Nature; ZMMU = Zoological Museum of Moscow State University. *Topotype.

Sample no.	Species	Voucher no.	Locality	GenBank acc. no.	Source
**Ingroup**					
1	*Tylototritonsoimalai* sp. nov.	CUMZ-A-8253	Mae Tuen Wildlife Sanctuary, Tak, Thailand	PQ218721	This study
2	*Tylototritonsoimalai* sp. nov.	CUMZ-A-8254	Mae Tuen Wildlife Sanctuary, Tak, Thailand	PQ218722	This study
3	*Tylototritonsoimalai* sp. nov.	CUMZ-A-8256	Mae Tuen Wildlife Sanctuary, Tak, Thailand	PQ218723	This study
4	Tylototritonumphangensis*	CUMZ-A-8243	Umphang Wildlife Sanctuary, Tak, Thailand	OK092618	Pomchote et al. (2021b)
5	Tylototritonuyenoi*	KUHE 19147	Doi Suthep, Chiang Mai, Thailand	AB830733	Nishikawa et al. (2013a)
6	Tylototritonphukhaensis*	CUMZ-A-7719	Doi Phu Kha National Park, Nan, Thailand	MN912575	Pomchote et al. (2020b)
7	Tylototritonanguliceps*	VNMN A.2014.3	Muong Nhe, Dien Bien, Vietnam	LC017832	Le et al. (2015)
8	Tylototritonverrucosus*	KIZ 201306055	Husa, Yunnan, China	AB922818	Nishikawa et al. (2014)
9	Tylototritonpanhai*	No voucher	Phu Luang Wildlife Sanctuary, Loei, Thailand	AB830736	Nishikawa et al. (2013a)
10	Tylototritonshanjing*	NMNS 3682	Jingdong, Yunnan, China	AB830721	Nishikawa et al. (2013a)
11	*Tylototritonpulcherrimus*	KUHE 46406	Yunnan, China	AB830738	Nishikawa et al. (2013a)
12	*Tylototritonpodichthys*	KUHE 34399	Xam Neua, Houa Phan, Laos	AB830727	Nishikawa et al. (2013a)
13	Tylototritonpanwaensis*	CAS 245418	Panwa, Myitkyina, Myanmar	KT304279	Grismer et al. (2018)
14	*Tylototritonyangi*	KUHE 42282	Yunnan, China	AB769546	Nishikawa et al. (2013b)
15	Tylototritonshanorum*	CAS 230940	Taunggyi, Shan, Myanmar	AB922823	Nishikawa et al. (2014)
16	*Tylototritonhimalayanus*	MVZ no number	Nepal	DQ517854	Weisrock et al. (2006)
17	Tylototritonkachinorum*	ZMMU A5953	Indawgyi, Kachin, Myanmar	MK097273	Zaw et al. (2019)
18	*Tylototritonkweichowensis*	MVZ 230371	Daguan, Yunnan, China	DQ517851	Weisrock et al. (2006)
19	*Tylototritontaliangensis*	KUHE 43361	Unknown, China	AB769543	Nishikawa et al. (2013b)
**Outgroup**					
20	Echinotritonandersoni*	KUHE no number	Nago, Okinawa, Japan	AB769545	Nishikawa et al. (2013b)

### ﻿Morphological examination

Morphometric comparisons and morphological differences between the Mae Tuen WS newts and *T.uyenoi* and *T.umphangensis* were examined using data from Pomchote et al. (2020a) for *T.uyenoi* and from Pomchote et al. (2021b) for *T.umphangensis* for three reasons. Firstly, the color pattern is rather similar between the Mae Tuen WS population and the two chosen *Tylototriton* species. Secondly, the genetic sequence divergences between the Mae Tuen WS newts and the two *Tylototriton* species were found to be lower than those observed in other pairs between Mae Tuen WS newts and the other species (%; as reported in this study). Thirdly, the distribution of the Mae Tuen WS population in the northeastern region (Fig. 1, locality 14) overlaps with the distribution ranges of *T.uyenoi* in its northern (Fig. 1, locality 11) and western (Fig. 1, localities 12 and 13) range, and with *T.umphangensis* in its western range (Fig. 1, locality 15). Moreover, previous studies identified a male newt found at Mae Tuen WS as *T.uyenoi* (Hernandez 2017). Note that the other four *Tylototriton* species present in Thailand (*T.verrucosus*, *T.anguliceps*, *T.phukhaensis*, and *T.panhai*) were not included in this morphometric study for two reasons. Firstly, the external morphology of *T.verrucosus*, *T.anguliceps*, and *T.phukhaensis* are clearly different from that of *Tylototriton* sp. from Mae Tuen WS (see details in Pomchote et al. 2020a, 2020b, 2022); however, morphological comparisons using the published literature were undertaken, as detailed in the morphological comparisons below. Secondly, *T.panhai* has a different color pattern from those of the other Thai *Tylototriton* species. It has a dark ground coloration, with the exception of the dorsal head, upper and lower lips, parotoids, vertebral ridge, rib nodules, tips of fingers and toes, margins of the cloacal slit, and the dorsal and ventral edges of the tail, which are yellow, orange, or reddish brown. In contrast, the other Thai *Tylototriton* species exhibit bright color markings on the head, dorsum, tail, or sides of the body (Nishikawa et al. 2013a; Phimmachak et al. 2015; Hernandez and Pomchote 2020a; Pomchote et al. 2020a, 2020b, 2021b, 2022; this study). Moreover, *T.panhai* is a member of the subgenus Yaotriton (Nishikawa et al. 2013a; Dufresnes and Hernandez 2022), while *Tylototriton* sp. from Mae Tuen WS is assigned to the subgenus Tylototriton (this study).

A total of 17 male specimens, including the three *Tylototriton* sp. from Mae Tuen WS (CUMZ-A-8253, -8254, and -8256) of this study, plus four specimens of *T.umphangensis* [using data from Pomchote et al. (2021b)] and ten of *T.uyenoi* [using data from Pomchote et al. (2020a)] were used for the morphometric comparison.

The 27 measurements taken for morphometric comparison followed Pomchote et al. (2020a), where the character definitions followed Nishikawa et al. (2011): **SVL** (snout-vent length); **HL** (head length); **HW** (head width); **MXHW** (maximum head width); **SL** (snout length); **LJL** (lower jaw length); **ENL** (eyelid-nostril length); **IND** (internarial distance); **IOD** (interorbital distance); **UEW** (upper eyelid width); **UEL** (upper eyelid length); **OL** (orbit length); **AGD** (axilla-groin distance); **TRL** (trunk length); **TAL** (tail length); **VL** (vent length); **BTAW** (basal tail width); **MTAW** (medial tail width); **BTAH** (basal tail height); **MXTAH** (maximum tail height); **MTAH** (medial tail height); **FLL** (forelimb length); **HLL** (hind limb length); **2FL** (second finger length); **3FL** (third finger length); **3TL** (third toe length); and **5TL** (fifth toe length). All measurements were taken to the nearest 0.01 mm using a digital sliding caliper, and subsequently rounded to 0.1 mm. The **BWs** were recorded using a digital weighing scale to the nearest 0.1 gm.

The SVL, BW, and the other 26 ratio values to SVL (presented as the % SVL) were compared among the three Thai *Tylototriton* species. Due to the paucity of specimens, we did not conduct statistical tests. The relationships of all morphometric characters were examined using principal component analysis (PCA). All statistical analyses were performed using the SPSS v. 28 for Windows software.

For morphological comparisons, the data of the other related congeners were taken from previous works (Anderson 1871; Fang and Chang 1932; Nussbaum et al. 1995; Hou et al. 2012; Nishikawa et al. 2013a, 2014; Khatiwada et al. 2015; Le et al. 2015; Phimmachak et al. 2015; Fei and Ye 2016; Grismer et al. 2018, 2019; Zaw et al. 2019; Pomchote et al. 2020a, 2020b, 2021b, 2022; Dufresnes and Hernandez 2022; Decemson et al. 2023).

The skulls of each specimen from the new species of this study (CUMZ-A-8253), *T.umphangensis* [CUMZ-A-8246, details of this specimen were reported in Pomchote et al. (2021b)], and *T.uyenoi* [THNHM 13866, loaned from the Natural History Museum, National Science Museum, Thailand (**THNHM**) —details of this specimen were provided in Pomchote et al. (2021b)] were scanned using micro X-ray computerized tomography (micro-CT; Bruker SkyScan 1173; 80 kV and 100 A; distortion-free Flat Panel sensor 2,240 × 2,240 pixels). This scanning process was conducted at the Scientific and Technological Research Equipment Center (STREC), Chulalongkorn University, Thailand. The segmentation and 3D reconstruction were performed using 3D Slicer v. 4.11.20200930 (Fedorov et al. 2012).

## ﻿Results

### ﻿Molecular analyses

We obtained 452–1,035 bp sequences of the partial ND2 gene region for 20 specimens, including the outgroup (Table 1). The sequences of the three specimens from Mae Tuen WS (this study) were the same, and of the 2,804 nucleotide sites, 451 were variable and 158 were parsimony informative within the ingroup (sequence statistics available upon request from the senior author). The mean likelihood score of the BI analyses for all trees sampled at stationary was −7689.148. The likelihood value of the ML tree was −7652.488946.

**Figure 3.**
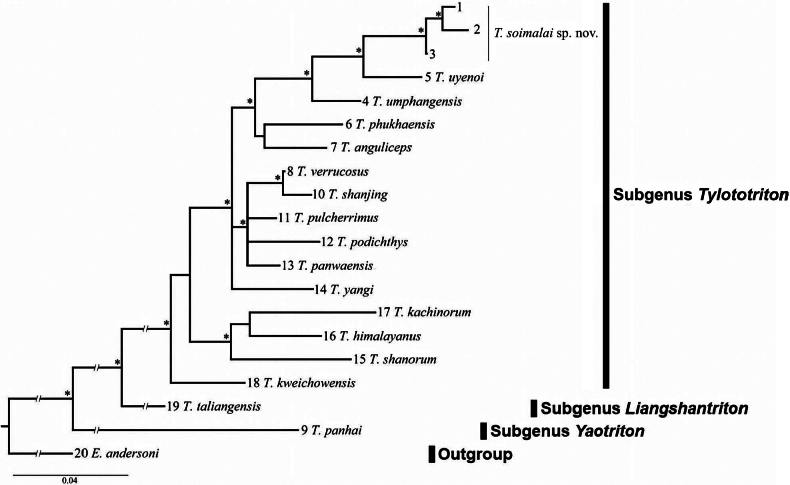
Bayesian inference tree based on the partial ND2 gene for the samples examined. Asterisks indicate nodes with bpp ≥ 0.95 and bs ≥ 70%. Numbers at branch tips are the sample numbers, as shown in Table 1. Scale bar: 0.04 substitutions/site.

Phylogenetic analyses employing the BI and ML criteria yielded nearly identical topologies and so we present only the BI tree in Fig. 3. Monophyly of the subgenus Tylototriton was fully supported in the BI and ML trees (bpp = 98% and bs = 96%). Within this subgenus, *T.kweichowensis* was first separated from the remaining lineages. The latter group was further divided into two clades: one including *T.shanorum*, *T.himalayanus*, and *T.kachinorum*; the other included the remaining lineages. The newts from Mae Tuen WS were nested in the latter clade and were clustered with *T.uyenoi* with significant support.

The *p*-distances between each pair of a total 19 haplotypes recognized above ranged from 0.8% (between *Tylototriton* sp. specimens) to 11.7% (between *T.uyenoi* and *T.kachinorum*) (Table 2). The distance between the newts from Mae Tuen WS and its sister species *T.uyenoi* was 4.1–4.2%, which was less than and/or comparable (4.2% between *T.anguliceps* and *T.verrucosus*, and 4.3% between *T.anguliceps* and *T.phukhaensis*) to the 17 heterospecific combinations in this study.

**Table 2.** Uncorrected *p*-distance (%) of the ND2 region between samples examined in this study.

Sample no.	Species	Sample no.																		
		1	2	3	4	5	6	7	8	9	10	11	12	13	14	15	16	17	18	19
**1**	*Tylototritonsoimalai* sp. nov.																			
**2**	*Tylototritonsoimalai* sp. nov.	0.010																		
**3**	*Tylototritonsoimalai* sp. nov.	0.008	0.010																	
**4**	*Tylototritonumphangensis*	0.057	0.058	0.057																
**5**	*Tylototritonuyenoi*	0.041	0.042	0.041	0.049															
**6**	*Tylototritonphukhaensis*	0.080	0.083	0.070	0.060	0.072														
**7**	*Tylototritonanguliceps*	0.068	0.069	0.068	0.050	0.073	0.043													
**8**	*Tylototritonverrucosus*	0.072	0.073	0.072	0.048	0.071	0.048	0.042												
**9**	*Tylototritonpanhai*	0.137	0.138	0.137	0.139	0.142	0.128	0.130	0.124											
**10**	*Tylototritonshanjing*	0.075	0.076	0.075	0.053	0.075	0.054	0.045	0.009	0.124										
**11**	*Tylototritonpulcherrimus*	0.067	0.068	0.067	0.055	0.068	0.044	0.040	0.019	0.120	0.025									
**12**	*Tylototritonpodichthys*	0.081	0.082	0.081	0.070	0.084	0.058	0.050	0.035	0.123	0.039	0.033								
**13**	*Tylototritonpanwaensis*	0.076	0.079	0.066	0.054	0.076	0.053	0.044	0.022	0.126	0.031	0.026	0.034							
**14**	*Tylototritonyangi*	0.075	0.076	0.075	0.056	0.075	0.056	0.042	0.038	0.126	0.045	0.038	0.049	0.041						
**15**	*Tylototritonshanorum*	0.095	0.096	0.095	0.083	0.093	0.078	0.068	0.062	0.124	0.066	0.066	0.074	0.066	0.068					
**16**	*Tylototritonhimalayanus*	0.091	0.095	0.080	0.079	0.086	0.067	0.067	0.062	0.123	0.061	0.063	0.067	0.064	0.065	0.052				
**17**	*Tylototritonkachinorum*	0.111	0.111	0.111	0.088	0.117	0.086	0.077	0.071	0.133	0.080	0.077	0.080	0.064	0.082	0.077	0.053			
**18**	*Tylototritonkweichowensis*	0.083	0.085	0.072	0.077	0.081	0.058	0.060	0.053	0.106	0.056	0.052	0.056	0.052	0.060	0.062	0.060	0.064		
**19**	*Tylototritontaliangensis*	0.097	0.098	0.097	0.093	0.100	0.086	0.085	0.073	0.106	0.070	0.072	0.078	0.077	0.078	0.083	0.076	0.073	0.063	
**20**	*Echinotritonandersoni*	0.181	0.180	0.181	0.172	0.181	0.168	0.166	0.159	0.155	0.161	0.157	0.161	0.149	0.160	0.159	0.157	0.188	0.152	0.148

### ﻿Morphological examination

A total of 17 adult males were used for the morphometric comparisons and morphological differences, as shown in Table 3 and Table 4, respectively. The Mae Tuen WS samples, *T.uyenoi*, and *T.umphangensis* showed some similar morphological characteristics. For instance, the nostrils were visible from the dorsal view, the surface of the dorsolateral bony ridges was rough, the glandular skin was dense, particularly on the dorsum, and the tips of the fore- and hind limbs overlapped when adpressed along the body. However, there were morphological differences between the Mae Tuen WS samples and the other two *Tylototriton* species. For example, in lateral view, the dorsolateral bony ridges of the Mae Tuen WS population were oriented rather parallel to body axis, while those of *T.uyenoi* and *T.umphangensis* were oriented obliquely upwards and curved upwardly at the posterior end. The vertebral ridge of the Mae Tuen WS population was not segmented, while those of *T.uyenoi* and *T.umphangensis* were segmented (see details in Table 4). Regarding coloration, they displayed rather similar color patterns. In life, *T.uyenoi* had a dark brown to black color background, while the Mae Tuen WS samples and *T.umphangensis* were black. The dorsal and ventral head, parotoids, vertebral ridge, rib nodules, limbs, vent, and tail were orange-brown in *T.umphangensis*, being dark orange-brown in *T.uyenoi*, but the Mae Tuen WS samples had a somewhat paler orange coloration. The ventral tail ridge had the brightest coloration among these three congeners. In preservative, the color pattern of *T.umphangensis* remained relatively similar to that observed in life after approximately two years. However, the background color of the *Tylototritonsoimalai* sp. nov. samples was blackish brown, and the color markings shifted to a paler orange hue after one year in preservative.

**Table 3.** Morphometric comparisons of the examined specimens of *Tylototriton* [median SVL (in mm), BW (in g), and ratios of characters (R: % SVL), with the range in parentheses]. Data for *T.uyenoi* are derived from Pomchote et al. (2020a) and for *T.umphangensis* are derived from Pomchote et al. (2021b). For character abbreviations refer to the text.

	*T.soimalai* sp. nov.	*T.umphangensis*	*T.uyenoi*
	3 males	4 males	10 males
SVL	66.5 (66.3–66.5)	73.5 (65.6–75.3)	71.1 (68.9–75.8)
BW	12.2 (10.8–12.7)	12.4 (10.2–13.3)	15.1 (11.2–17.0)
RHL	27.2 (27.1–27.4)	23.0 (22.0–25.2)	25.1 (24.2–26.3)
RHW	21.7 (21.4–23.0)	21.4 (19.4–22.7)	18.8 (17.5–19.3)
RMXHW	24.7 (24.4–24.9)	25.6 (25.0–26.9)	25.8 (24.5–26.4)
RSL	9.4 (8.2–9.5)	8.8 (8.2–9.8)	8.8 (8.1–9.4)
RLJL	24.3 (23.6–24.3)	22.8 (22.1–23.5)	22.0 (20.7–22.5)
RENL	5.9 (5.2–6.1)	5.8 (5.6–6.2)	6.8 (6.0–7.5)
RIND	5.5 (5.2–5.6)	6.2 (5.8–6.5)	6.8 (5.6–7.5)
RIOD	13.3 (12.7–13.7)	13.2 (12.9–13.7)	13.0 (12.6–14.4)
RUEW	3.5 (3.1–3.7)	2.5 (2.3–2.9)	3.1 (2.2–3.8)
RUEL	7.1 (6.3–7.3)	6.0 (5.5–6.4)	6.4 (5.8–7.1)
ROL	4.0 (3.7–4.3)	3.0 (2.7–3.3)	4.2 (3.5–4.8)
RAGD	50.6 (50.4–57.3)	53.7 (51.9–54.4)	49.9 (45.7–52.3)
RTRL	76.5 (74.1–80.0)	76.8 (75.4–77.4)	75.0 (71.8–98.0)
RTAL	101.5 (90.7–109.3)	104.7 (91.9–107.3)	98.0 (88.8–110.4)
RVL	9.7 (6.6–9.9)	8.0 (7.3–9.4)	12.4 (7.4–15.3)
RBTAW	12.1 (11.0–14.1)	14.5 (12.6–15.1)	5.8 (4.4–6.2)
RMTAW	3.0 (2.9–3.8)	2.3 (2.2–2.4)	3.7 (2.9–4.3)
RBTAH	11.9 (11.6–13.8)	15.0 (11.9–15.3)	12.1 (11.5–12.9)
RMXTAH	15.5 (14.6–16.7)	11.1 (8.8–12.1)	12.7 (11.0–14.2)
RMTAH	15.9 (14.2–16.2)	10.6 (7.9–12.0)	11.8 (11.0–13.3)
RFLL	41.4 (38.2–41.8)	37.0 (34.2–40.5)	43.7 (42.6–44.6)
RHLL	39.1 (37.1–39.5)	38.4 (35.2–41.9)	44.8 (42.3–48.1)
R2FL	6.3 (5.9–7.0)	7.1 (6.7–8.1)	5.5 (4.5–6.8)
R3FL	6.7 (6.5–7.6)	7.6 (5.6–8.9)	6.8 (5.5–7.4)
R3TL	9.5 (7.2–9.6)	9.3 (8.9–11.0)	8.3 (7.0–9.2)
R5TL	3.4 (2.8–4.4)	4.9 (4.8–5.7)	4.1 (2.8–6.1)

**Table 4.** Morphological comparisons between *Tylototritonsoimalai* sp. nov. and *T.umphangensis* and *T.uyenoi*. Data for *T.uyenoi* are modified from Pomchote et al. (2020a), and *T.umphangensis* are modified from Pomchote et al. (2021b).

Characters	*T.soimalai* sp. nov.	*T.umphangensis*	*T.uyenoi*
Number and sex	3 males	4 males	10 males
Snout in dorsal view	Blunt or truncate	Truncate	Rounded to blunt
Snout in lateral view	Projecting beyond lower jaw	Hardly projecting beyond lower jaw	Projecting beyond lower jaw
Sagittal ridge	Narrow, short	Wide	Wide
Dorsolateral bony ridges in dorsal view	Weakly or hardly curved medially at posterior end	Distinctly curved medially at posterior end	Weakly or rather curved medially at posterior end
Dorsolateral bony ridges in lateral view	Oriented rather parallel to body axis	Oriented obliquely upwards and curved upwardly at posterior end	Oriented obliquely upwards and curved upwardly at posterior end
Parotoids in lateral view	Oriented rather parallel to body axis and slightly or hardly curved upwardly at posterior end	Oriented rather parallel to body axis and curved upwardly at posterior end	Oriented obliquely downwardly or rather parallel to body axis and not curved or curved upwardly at posterior end
Vertebral ridge	Not segmented	Segmented	Segmented
Rib nodules	Distinct, rounded, isolated but connected posteriorly, 14–16	Indistinct, rounded anteriorly to irregularly shaped posteriorly, isolated, 14–15	Distinct, rounded, isolated, 12–16

**Figure 4.**
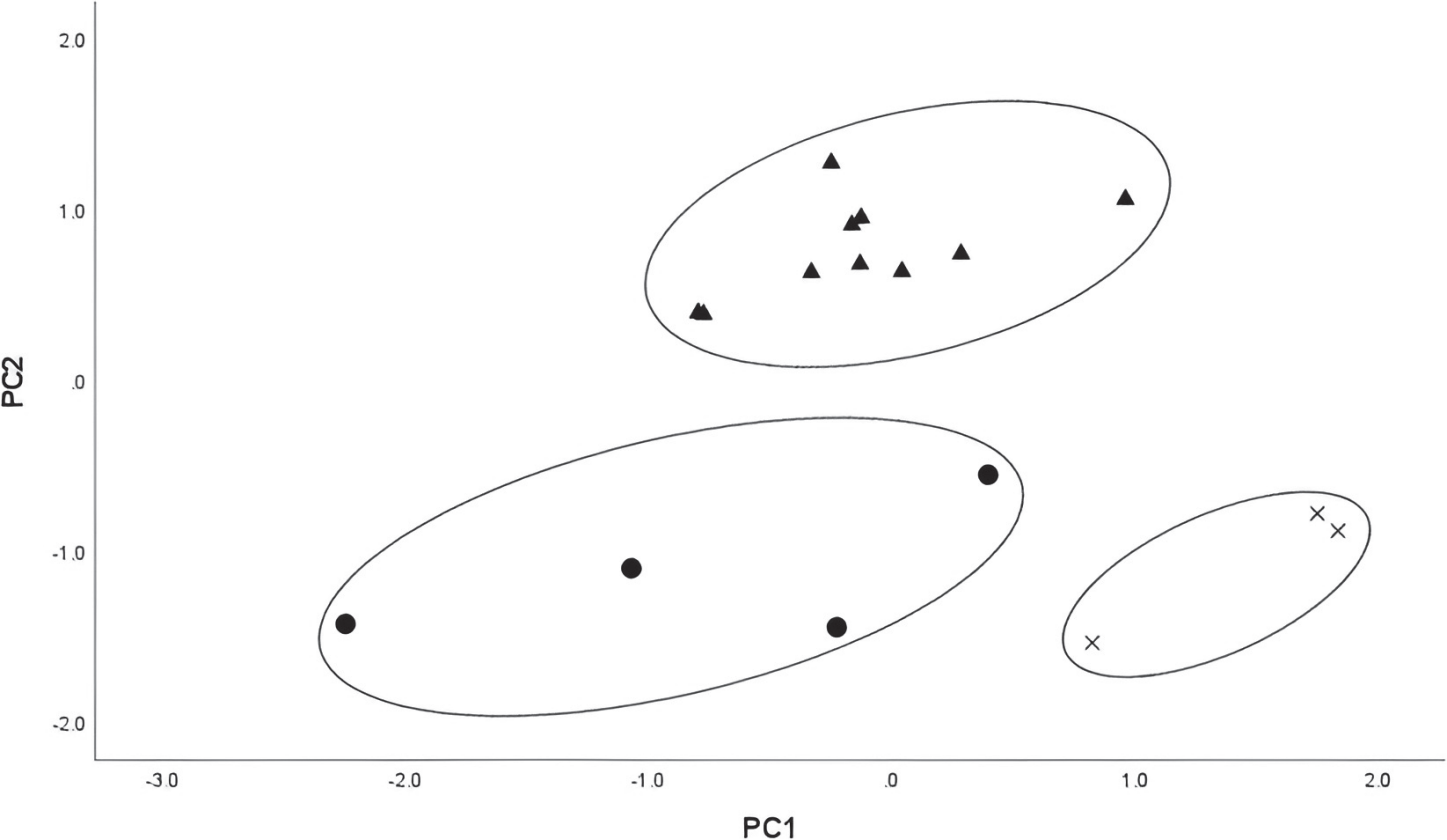
The PCA plots of PC1 versus PC2 for morphological parameters of the samples examined: **cross***Tylototritonsoimalai* sp. nov. **circle***T.umphangensis***triangle***T.uyenoi*.

The overall morphological differences between the *Tylototritonsoimalai* sp. nov. population and the other two *Tylototriton* species included in the morphological study were examined using PCA. The first two principal components (PCs) accounted for 49.4% of the total variation. The two-dimensional PC1 vs PC2 plot showed that the *Tylototritonsoimalai* sp. nov. population was clustered together and completely separated from its closely related species, *T.uyenoi* and *T.umphangensis* (Fig. 4).

**Figure 5.**
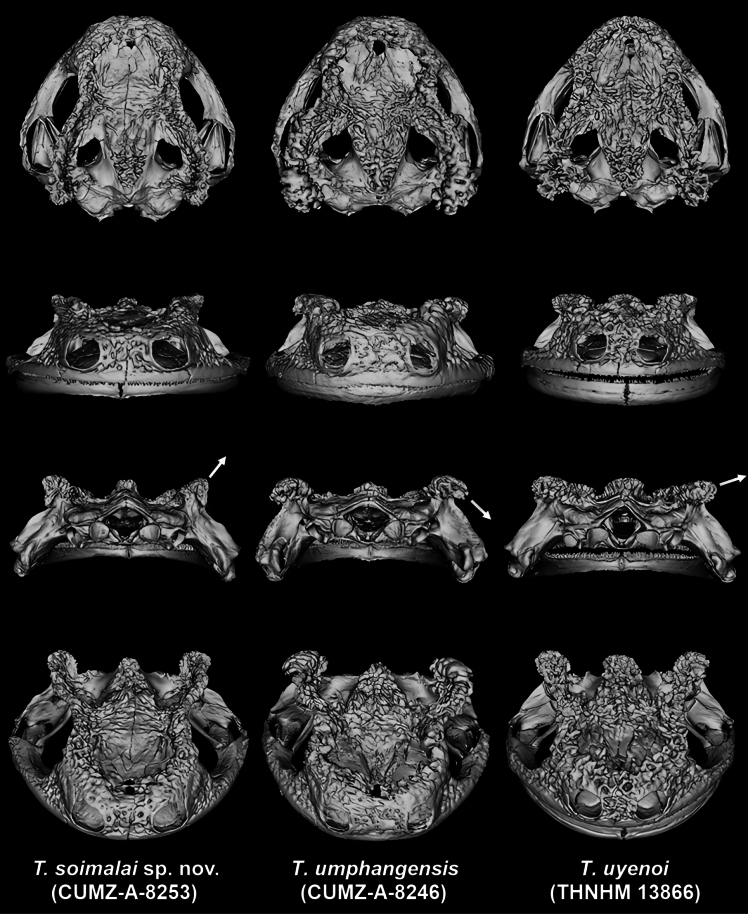
Three-dimensional model of the skull of *Tylototritonsoimalai* sp. nov. (left), *T.umphangensis* (center), and *T.uyenoi* (right) based on micro-CT reconstruction. **Top** dorsal view **Second from the top** anterior view **Second from the bottom** posterior view **Bottom** anteriodorsal view. White arrows representing directions of posterior ends of dorsolateral bony ridges.

There are some differences in the skull morphology among *Tylototritonsoimalai* sp. nov., *T.uyenoi*, and *T.umphangensis* (Fig. 5). For example, the distance between the nostrils was widest in *T.umphangensis*, followed by *Tylototritonsoimalai* sp. nov. and *T.uyenoi*, respectively. The midsagittal ridge was more distinct in *Tylototritonsoimalai* sp. nov. compared to *T.uyenoi* and *T.umphangensis*. Additionally, the density of secondary bony ridges was greater in *T.uyenoi* and *T.umphangensis* compared to *Tylototritonsoimalai* sp. nov. However, the most prominently different character among the skull of the three species was the direction of the posterior ends of the dorsolateral bony ridges in the posterior view. In *Tylototritonsoimalai* sp. nov., they were directed more upwards than those of *T.uyenoi*, while in *T.umphangensis*, they were directed downwards.

Based on the molecular and morphological evidence, we herein describe the *Tylototriton* sp. from Mae Tuen WS, Tak Province, northwestern Thailand as a new species, *Tylototritonsoimalai* sp. nov.

### ﻿Systematics

#### ﻿ Tylototriton soimalai sp. nov.

Taxon classificationAnimaliaUrodelaSalamandridae

9D8C6639-9101-5D74-9FE7-031702BEB7BF

https://zoobank.org/8CB99CD2-A029-4FD4-928C-82AD853F9A04

**Figure 6.**
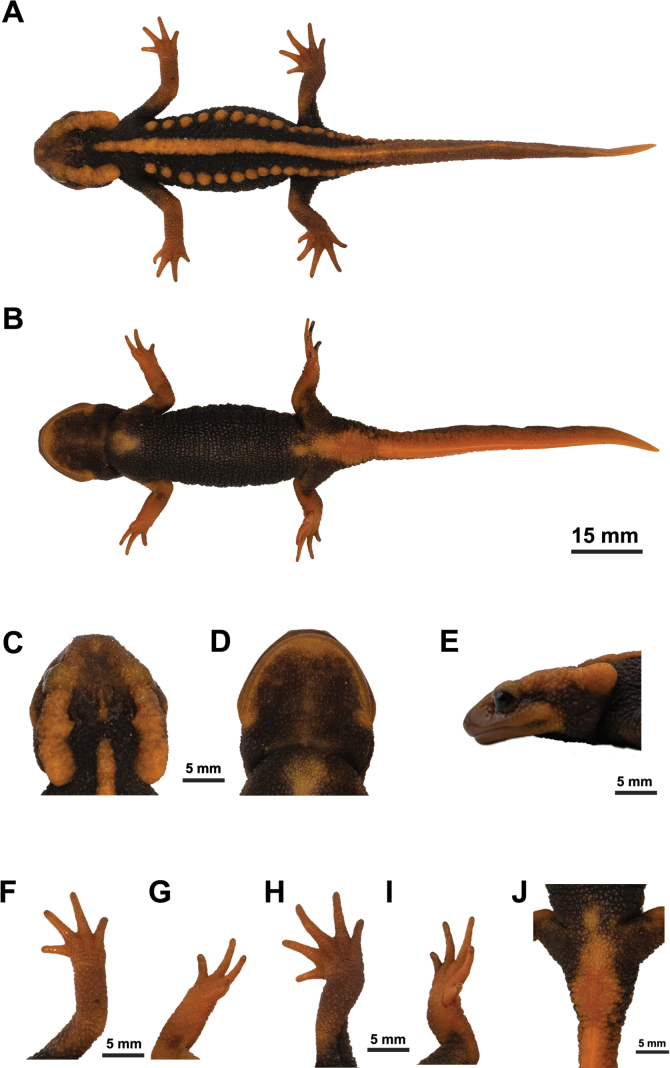
The male holotype of *Tylototritonsoimalai* sp. nov. (CUMZ-A-8253) before preservation **A** dorsal view **B** ventral view **C** dorsal head **D** ventral head **E** lateral head **F** dorsal right hand **G** ventral right hand **H** dorsal right foot **I** ventral right foot **J** cloacal area.

**Figure 7.**
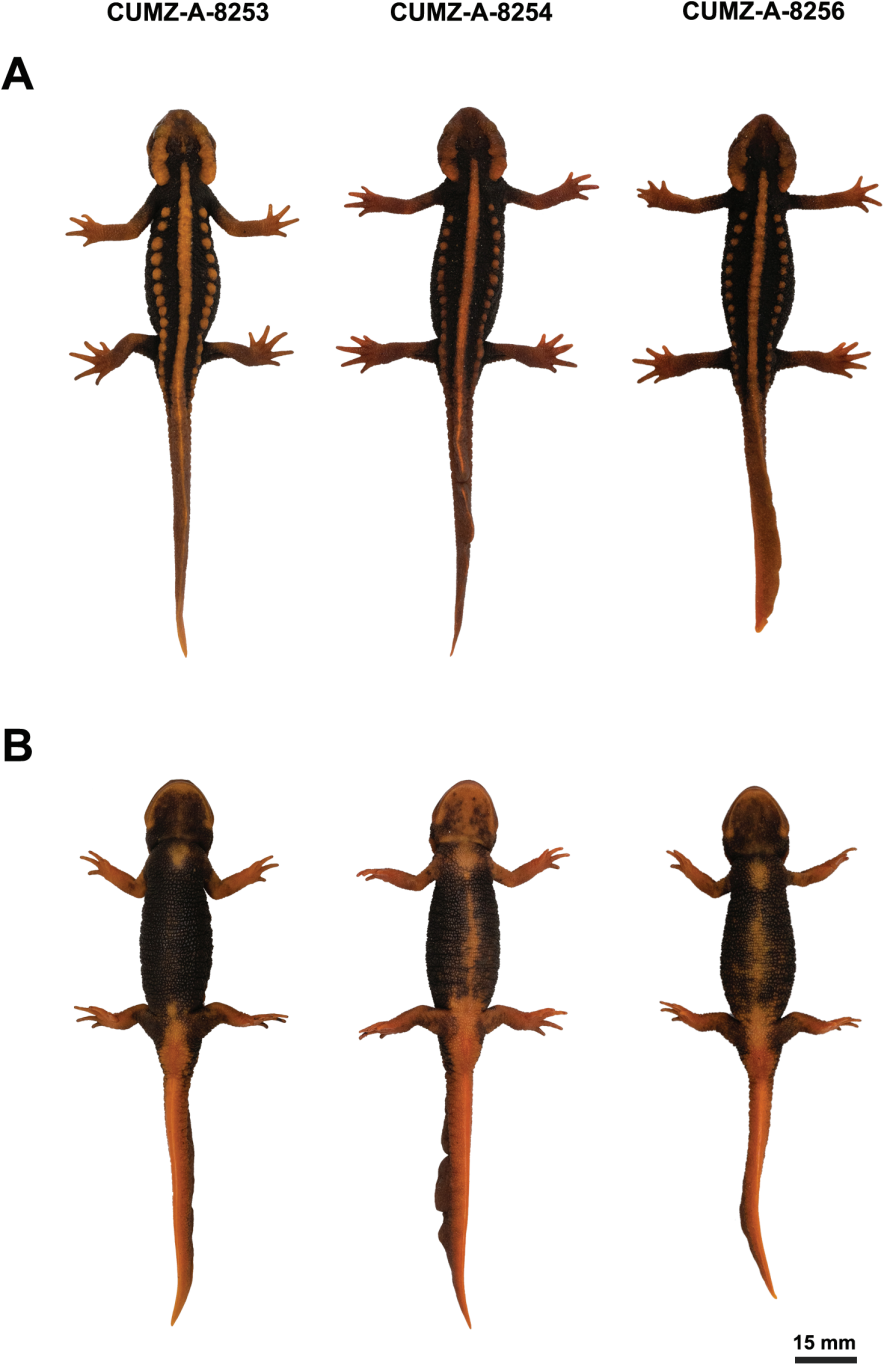
Holotype (CUMZ-A-8253) and paratypes (CUMZ-A-8254 and CUMZ-A-8256) of *Tylototritonsoimalai* sp. nov. before preservation **A** dorsal view **B** ventral view.

**Figure 8.**
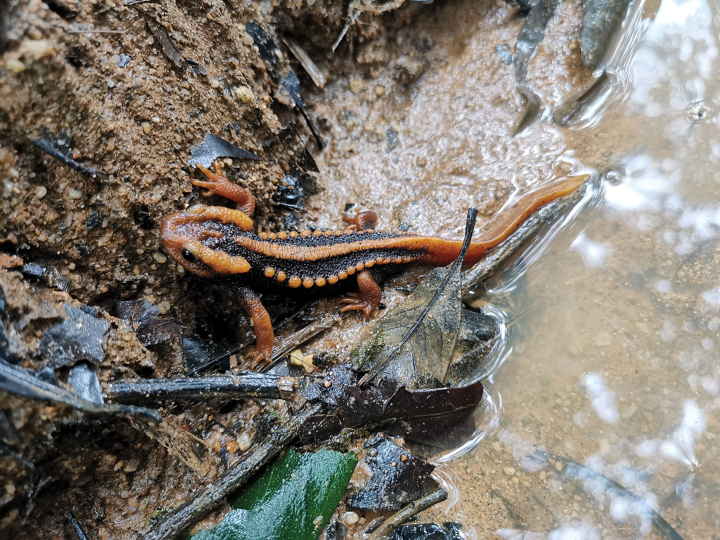
The male holotype of *Tylototritonsoimalai* sp. nov. (CUMZ-A-8253) observed at the type locality.

Figs 2 , 5 , 6 , 7 , 8 (Thai name: Ka Tang Nam Doi Soi Malai) (English name: Doi Soi Malai Crocodile Newt)

Tylototriton uyenoi : Hernandez (2017): 110.

##### Type material.

***Holotype*** • CUMZ-A-8253, adult male, collected from Doi Soi Malai, Mae Tuen Wildlife Sanctuary, Tak Province, northwestern Thailand, at ca 1,500 m a.s.l., collected on the 31 August 2022 by Porrawee Pomchote and Pitak Sapewisut. Data regarding the specific location (geographical coordinates) of the new species cannot be publicly disclosed due to the need to prevent illegal hunting, which has been increasing dramatically in Thailand. However, the data are available to the editors or reviewers if necessary. ***Paratypes*** • CUMZ-A-8254 and CUMZ-A-8256; two adult males, same data as the holotype.

##### Etymology.

The specific epithet *soimalai* refers to Doi Soi Malai, Mae Tuen Wildlife Sanctuary, the type locality of the new species; it is a noun in apposition, thus invariable.

##### Diagnosis.

*Tylototritonsoimalai* sp. nov. is assigned to the genus *Tylototriton* by having a combination of dorsal granules present, dorsolateral bony ridges on head present, knob-like warts or rib nodules on dorsolateral body present, and quadrate spine absent. *Tylototritonsoimalai* sp. nov. is distinguished from its congeners by a combination of the following morphological characters: (1) medium-sized, adult SVL 66.3–66.5 mm in males; (2) skin rough with fine granules; (3) head longer than wide; (4) snout blunt or truncate in dorsal view, and extending beyond the lower jaw in lateral view; (5) sagittal ridge on head narrow, short, and distinct; (6) dorsolateral bony ridges on head pronounced, with rough surface, posterior ends weakly or hardly curved medially in dorsal view, and oriented rather parallel to the body axis in lateral view; (7) parotoids distinct, oriented rather parallel to the body axis and posterior ends slightly or hardly curved upwards in lateral view; (8) vertebral ridge prominent, wide, and not segmented; (9) rib nodules distinct, rounded, and isolated but posterior nodules connected, 14–16 along each side of body; (10) limbs long, tips of forelimbs and hind limbs overlapping when adpressed along body; (11) tail laterally compressed, lacking lateral grooves, and tip pointed; (12) background coloration black; (13) dorsal, ventral, and lateral of head, parotoids, vertebral ridge, rib nodules, limbs, vent region, and whole tail with orange markings.

##### Description of holotype.

Body slim and long (RTRL 80.0%); skin rough; fine granules dense on dorsum, dense on both sides of body and tail, and sparse on ventral trunk; head longer than wide (HW/HL 0.8), hexagonal in shape, depressed, and slightly oblique in profile; snout truncate in dorsal view, projecting beyond lower jaw in lateral view; eyes protrude from dorsolateral portion of head in dorsal view, and upper eyelids prominent in lateral view; nostrils close to snout tip, visible from dorsal view; sagittal ridge on head narrow, short, and distinct; dorsolateral bony ridges on head pronounced, rough, and posterior ends weakly curved proximally in dorsal view; labial fold absent; tongue oval, attached to anterior floor of mouth, free laterally and posteriorly; vomerine tooth series in an inverted V-shape, converging anteriorly, and reaching choanae; parotoids distinct, projecting posteriorly, posterior ends hardly curved medially in dorsal view, oriented rather parallel to body axis and hardly curved upwards in lateral view; gular fold present; costal folds absent; vertebral ridge prominent, wide, and not segmented, separated from sagittal ridge on head; rib nodules distinct, rounded, forming knob-like warts, 14 on left side and 16 on right side of body from axilla to base of tail; rib nodules isolated but posterior nodules connected; rib nodules slightly increasing in size from most anterior to third nodule, then decreasing posteriorly; forelimbs (41.8% SVL) longer than hind limbs (39.5% SVL); tips of forelimb and hind limb overlapping when adpressed along body; fingers and toes well developed, free of webbing; fingers four, comparative finger lengths 3 > 2 > 1 > 4; toes five, comparative toe lengths 3 > 4 > 2 > 5 > 1; tail laterally compressed, lacking lateral grooves, dorsal fin and ventral edge smooth, tip pointed; tail as long as body length (101.5% SVL); cloaca slightly swollen; vent slit longitudinal.

##### Color of holotype.

In life, dorsal ground coloration is black, while the ventral color is dark grayish, paler than dorsum. Dorsal, ventral, and lateral of head, parotoids, vertebral ridge, rib nodules, limbs, vent region, and whole tail are orange. Tip of tail is slightly paler than dorsal and lateral sides of tail. Ventral side of head, part of pectoral and pubic region, limbs, and tail are paler than dorsum. The palest is the ventral edge of the tail. The paler region between the ventral edge of the tail and the area of the vent is connected. After preservation in ethanol for approximately one year, the background color is blackish brown, and the color markings are faded to pale orange.

##### Measurement of holotype

**(in mm).**SVL 66.5; HL 18.0; HW 14.3; MXHW 16.6; SL 6.3; LJL 16.2; ENL 4.0; IND 3.7; IOD 8.9; UEW 4.2; UEL 2.1; OL 2.5; AGD 38.1; TRL 53.3; TAL 67.5; VL 6.5; BTAW 9.4; MTAW 2.5; BTAH 7.9; MXTAH 10.3; MTAH 10.6; FLL 27.8; HLL 26.3; 2FL 4.2; 3FL 5.1; 3TL 6.3; and 5TL 2.9.

##### Variation.

All specimens generally exhibit a similar morphology and coloration; however, some differences were observed among the three specimens. The snout of the holotype is truncate, while those of two paratypes (CUMZ-A-8254 and CUMZ-A-8256) are blunt. The sagittal ridge is most distinct in the holotype, followed by CUMZ-A-8256 and CUMZ-A-8254, respectively. Dorsolateral bony ridges of the holotype and one paratype (CUMZ-A-8254) weakly curve medially in dorsal view, in contrast to the other paratype where they hardly curve medially in dorsal view. The posterior ends of parotoids in two paratypes slightly curve upwards in lateral view compared to the holotype that hardly curve upwards in lateral view. Rib nodules of the holotype are 14 on the left side and 16 on the right side of the body from axilla to base of tail, while the two paratypes have 14 on left side and 15 on right side (CUMZ-A-8254) or 15 on both left and right sides (CUMZ-A-8256). One paratype (CUMZ-A-8256) has five fingers on the left forelimb; moreover, all finger lengths on the left forelimb are short. The dorsal tail fin is smooth in the holotype, whereas in the other paratypes it is uneven with CUMZ-A-8256 exhibiting the most pronounced unevenness. The color marking on the dorsal side of the holotype is the palest compared to the two paratypes. The palest color marking on the ventral side of the head is clearly observed in one paratype (CUMZ-A-8254) followed by CUMZ-A-8256 and the holotype, respectively. The part of the ventral trunk of two paratypes exhibits a pale color marking, whereas there is no pale color marking on the ventral trunk of the holotype.

##### Larva.

Two larvae one nearly double the size of the other (Fig. 2). Head large. Eyes well visible. Three pairs of external gills present. Body and tail laterally compressed. Skin smooth. Costal grooves of larger-sized larva rather distinct, but smaller-sized larva indistinct. Dorsal and ventral fins present. Background color of larger larva pale brown with scattered black pigments. Parts of lateral body, ventral fin, and around eyeballs silver-purple, while the smaller larva had a pale brown background with dense black pigments. Parts of lateral body, and around eyeballs silver-purple. External gills of both larvae red-brown.

##### Comparisons.

*Tylototritonsoimalai* sp. nov. is a member of the subgenus Tylototriton based on the molecular phylogenetic analyses. The new species can be distinguished from the other members of the subgenus Tylototriton as follows: from *T.anguliceps*, *T.phukhaensis*, *T.kachinorum*, and *T.shanorum* by having a narrow, short, and distinct sagittal ridge (vs prominent in *T.anguliceps*, narrow, long, and distinct in *T.phukhaensis*, very weak and almost indistinct in *T.kachinorum*, and absent in *T.shanorum*); from *T.verrucosus* and *T.podichthys* by having rough dorsolateral bony ridges (vs smooth in *T.verrucosus* and very rough in *T.podichthys*); from *T.zaimeng* by having an inverted V-shape of the vomerine tooth series (vs a bell-shape in *T.zaimeng*); from *T.panwaensis* by having a non-segmented vertebral ridge (vs weakly segmented in *T.panwaensis*); from *T.himalayanus* by lacking grooves on either side at the base of tail (vs present in *T.himalayanus*); from *T.shanjing* by having no sharp contrast between the orange crown of the head and black nape (vs sharp contrast in *T.shanjing*); from *T.yangi* by having uniformly orange parotoids (vs black coloration except for posterior end of parotoids with orange coloration in *T.yangi*); from *T.kweichowensis* by having isolated pale markings on rib nodules (vs connected markings forming continuous pale dorsolateral lines in *T.kweichowensis*); from *T.ngarsuensis* by having orange markings on parotoids, vertebral ridge, rib nodules, and limbs (vs dark-brown, nearly black coloration in *T.ngarsuensis*); from *T.houi* by having orange markings on the head, trunk, limbs, and tail (vs extensive orange-red markings in *T.houi*); and from *T.pulcherrimus* by lacking pale spots located ventrolaterally and on flanks (vs present in *T.pulcherrimus*).

##### Distribution.

*Tylototritonsoimalai* sp. nov. is currently known from only Doi Soi Malai, Mae Tuen Wildlife Sanctuary, Tak Province, northwestern Thailand. However, Doi Soi Malai-Mai Klay Pen Hin National Park, which is contiguous to Mae Tuen Wildlife Sanctuary, is also expected to be a habitat for this species.

##### Natural history.

The new species were found during the midday, at ~ 12:00 h when the adult males came up to the water surface, and the two larvae lived in a single isolated mud puddle situated along the road to the top of Doi Soi Malai during the rainy season, which is the breeding season of *Tylototriton* species. The puddle had turbid water and the bottom was deposited with muddy sediment. The surrounding area of the puddle consisted of evergreen hill forests. The puddle size was approximately 1,000 cm long, 500 cm wide, and 35 cm in maximum depth. No fish were observed.

##### Conservation recommendation.

The type locality of *Tylototritonsoimalai* sp. nov. is a well-known destination for mountain biking and 4×4 road trips, particularly in the period following the late rainy season, starting from October onwards, when these activities extend to the summit of Doi Soi Malai. Although, the Department of National Parks, Wildlife and Plant Conservation (DNP) has imposed a ban on motor races in Thai NPs and WSs (The Nation 2019), some mountain biking and 4×4 road trips continue to violate these regulations by entering the Mae Tuen Wildlife Sanctuary (Park rangers, personal communications), likely due to the paved road that runs through the sanctuary, providing easy access (Pattanavibool and Dearden 2002). This could have adverse effects on the population of this new species, particularly during the larval stage, because the breeding site we found in this study is situated along the road leading to the summit of Doi Soi Malai. In nature, the breeding season of Thai *Tylototriton* species is around the end of April to August during the monsoon season (Pomchote et al. 2008; Hernandez and Pomchote 2020a, 2021). Aquatic larvae of *Tylototriton* inhabit the breeding water for several months and undergo complete metamorphosis before the efts start to move to land (Hernandez 2016). Based on data from Thai *Tylototriton* species in their natural habitat, larvae were found in the water bodies from August to November in *T.panhai* (Hernandez and Pomchote 2020a), from August to December in *T.uyenoi* (Nishikawa et al. 2013a; Pomchote, unpublished data), in December in *T.verrucosus* (Pomchote, unpublished data), and from December to March in *T.anguliceps* (Pomchote, unpublished data). Therefore, the breeding site should not be disturbed by any anthropogenic activities, especially road disturbances. We strongly recommend that the road to the summit of Doi Soi Malai be opened only after the breeding site has dried up, or alternatively, the road should be accessible during the winter and dry seasons, with walking to the peak as the preferred option.

Based on our multiple surveys conducted across various locations at Mae Tuen Wildlife Sanctuary in all seasons, we encountered only a few newts during the most recent survey at a single location on the 31 August 2022, suggesting that the population of the new species is small. Moreover, in addition to the road disturbances mentioned earlier, both the areas surrounding and within Mae Tuen Wildlife Sanctuary have been heavily impacted by habitat alteration and deforestation, leading to forest fragmentation, primarily due to agricultural activities, especially cabbage cultivation (Pattanavibool and Dearden 2002). Due to the reasons mentioned above, we recommend that *Tylototritonsoimalai* sp. nov. be listed as Endangered (EN) [IUCN Red List criteria B1ab(iii)+2ab(iii)] and, a conservation plan is urgently needed for this new species.

## ﻿Discussion

The current study employed morphological evidence, including external morphology, morphometrics, skull morphology, and molecular data. Consequently, the newt population from Doi Soi Malai, Mae Tuen WS in Tak Province, northwestern Thailand, previously identified as *T.uyenoi* (Hernandez 2017), is described as a new species, *Tylototritonsoimalai* sp. nov. Although the *T.uyenoi* specimens generally exhibit similarity in their morphology and color pattern, they display some morphological variations (Nishikawa et al. 2013a; Pomchote et al. 2020a) that may lead to confusion, particularly when attempting identification based on a limited number of individuals and external morphological characters without considering skull morphology or genetic analysis. Thus, conducting field surveys at the previously surveyed locations, voucher specimen collection, and both morphological and molecular analyses are essential for clarifying the taxonomic status and distribution range of T.cf.uyenoi. This is particularly the case at Doi Mak Lang, Chiang Dao WS, and Doi Mon Jong at Chiang Mai Province (Fig. 1, localities 6, 8, and 11, respectively); Mae Wong NP at Kamphaeng Phet Province (Fig. 1, locality 12); and Khao Laem NP at Kanchanaburi Province (Fig. 1, locality 13). These efforts will also help to clarify the boundaries between *Tylototritonsoimalai* sp. nov., T.cf.uyenoi, and *T.umphangensis* in order to manage a future conservation plan.

In summary, the genus *Tylototriton* currently comprises 41 nominal species, with seven of them occurring in Thailand: *T.verrucosus*, *T.uyenoi*, *T.anguliceps*, *T.phukhaensis* from the northern region, *T.panhai* from the northern and northeastern regions, *Tylototritonsoimalai* sp. nov. from the northwestern region, and *T.umphangensis* from the western region (Fig. 1) (Nishikawa et al. 2013a; Le et al. 2015; Hernandez and Pomchote 2020a; Pomchote et al. 2020a, 2020b, 2021b, 2022; this study).

## Supplementary Material

XML Treatment for Tylototriton soimalai

## ﻿Additional information

### Author ORCIDs

Porrawee Pomchote https://orcid.org/0000-0003-1035-5553

Wichase Khonsue https://orcid.org/0000-0002-3058-6378

Axel Hernandez https://orcid.org/0000-0003-3720-2856

Chitchol Phalaraksh https://orcid.org/0000-0002-7662-4931

Kanto Nishikawa https://orcid.org/0000-0002-6274-4959

### Data availability

All of the data that support the findings of this study are available in the main text.
